# The prognostic value of health-related quality of life for early molecular response in patients with chronic myeloid leukemia: analysis of the GIMEMA-SUSTRENIM trial

**DOI:** 10.3389/fonc.2025.1645217

**Published:** 2025-08-27

**Authors:** Fabio Efficace, Laura Cannella, Isabella Capodanno, Rosaria Sancetta, Veronica Porcu, Monia Lunghi, Annalisa Biagi, Fausto Castagnetti, Giovanni Caocci, Thomas Baldi, Roberto Marasca, Massimiliano Bonifacio, Antonella Gozzini, Bruno Martino, Federica Sora, Peter E. Westerweel, Paola Fazi, Marco Vignetti, Fabrizio Pane, Massimo Breccia

**Affiliations:** ^1^ Italian Group for Adult Hematologic Diseases (GIMEMA), Data Center and Health Outcomes Research Unit, Rome, Italy; ^2^ Hematology Unit, Azienda Unità Sanitaria Locale-IRCCS, Reggio Emilia, Italy; ^3^ Hematology Unit, Dell'Angelo Hospital, Venezia-Mestre, Italy; ^4^ Hematology Unit, S. Eugenio Hospital, ASL Roma 2, Tor Vergata University, Rome, Italy; ^5^ Division of Hematology, Department of Translation Medicine, University of Eastern Piedmont, Novara, Italy; ^6^ Hematology Unit, Santa Maria Goretti Hospital, Latina, Italy; ^7^ Institute of Hematology "Seràgnoli" IRCCS Azienda Ospedaliero-Universitaria di Bologna, Department of Medical and Surgical Sciences University of Bologna, Bologna, Italy; ^8^ Hematology Unit, Businco Hospital, Department of Medical Sciences and Public Health, University of Cagliari, Cagliari, Italy; ^9^ Section of Hematology, Department of Medical and Surgical Sciences, University of Modena and Reggio Emilia, Azienda Ospedaliero-Universitaria di Modena, Modena, Italy; ^10^ Department of Medicine, Azienda Ospedaliera Universitaria Integrata di Verona, Verona, Italy; ^11^ Department of Engineering for Innovation Biomedicine, Section of Innovation Biomedicine, Hematology Area, University of Verona, Verona, Italy; ^12^ Hematology Unit, AOU Careggi, University of Florence, Florence, Italy; ^13^ Division of Hematology, Grande Ospedale Metropolitano 'Bianchi Melacrino Morelli', Reggio Calabria, Italy; ^14^ Dipartimento di Diagnostica per Immagini, Radioterapia Oncologica ed Ematologia, Fondazione Policlinico Universitario A. Gemelli IRCCS, Università Cattolica Sacro Cuore, Rome, Italy; ^15^ Department of Internal Medicine, Albert Schweitzer Hospital, Dordrecht, Netherlands; ^16^ Hematology Section, Department of Clinical Medicine and Surgery, University of Naples "Federico II", Naples, Italy; ^17^ Hematology, Department of Translational and Precision Medicine, Azienda Ospedaliera Policlinico Umberto I, Sapienza University of Rome, Rome, Italy

**Keywords:** quality of life, patient reported outcome (PRO), functioning, chronic myeloid leukemia (CML), early molecular response (EMR)

## Abstract

We aimed to explore the prognostic value of patient-reported health-related quality of life (HRQoL) data for the achievement of early molecular response (EMR) at 3 months in patients with chronic phase chronic myeloid leukemia (CP-CML). We analyzed HRQoL baseline data of 436 newly diagnosed patients with CML patients enrolled in the GIMEMA Sustrenim trial. HRQoL was assessed by the EORTC QLQ-30 and the QLQ-CML24 questionnaires. In the multivariate analysis, the following factors were found to be independently associated with achievement of EMR: Sokal risk (low vs intermediate risk p=0.046 and low vs high risk p<0.001), nilotinib treatment (p<0.001) and higher patient-reported role functioning (EORTC QLQ-C30) (p<0.001). Current findings suggest the importance of assessing HRQoL at diagnostic workup of patients with CML as it may provide valuable prognostic information.

## Introduction

Tyrosine kinase inhibitors (TKIs) have improved the outcome of chronic myeloid leukemia patients (CML) with faster reduction of the burden of disease. Robust and standardized definitions of molecular responses have been introduced and are now universally accepted (1). Early molecular monitoring has become particularly useful to identify patients who might be considered for a prompt change of treatment. First evidence correlated with the early reduction of the molecular burden with the cytogenetic response (2, 3) and the lack of disease progression (3, 4). The cut-off of nearly 10% achieved within the first 3 months was first identified by Marin et al. (5) as predictive of better overall survival (OS). The same level of response, namely the early molecular response or EMR (BCR::ABL1 ratio ≤ 10% at 3 months) was then replicated in other studies as always associated with better OS, event-free survival (EFS) and subsequent increased possibility to achieve in the long-term a deep molecular response (BCR::ABL1 ratio ≤ 0.0032% or MR4.5) (6–8). The significance of EMR was then validated also with second generation TKIs, dasatinib and nilotinib, when tested as frontline treatment in two sponsored clinical trials (9, 10).

Previous work across several cancer populations (11, 12) has shown that health-related quality of life (HRQoL) data provides independent prognostic information for survival and, some evidence in the CML setting, indicated that HRQoL data may also predict achievement of a major molecular response (13). Therefore, leveraging from an international trial (14), we explored the prognostic value of baseline HRQoL data for the achievement of a BCR::ABL1 transcript level of ≤10% after 3 months of TKI treatment, being a key milestone of patients management. A Secondary objective was to describe the baseline HRQoL profile by frequently used prognostic scores: Sokal, EUTOS and ELTS.

## Patients and methods

The SUSTRENIM trial (Clinical Trial number 02602314), was a prospective, interventional, randomized, two arms, study in newly diagnosed chronic phase CML (CP-CML) patients treated with a second generation TKI (Nilotinib, NIL) or with Imatinib (IM) followed by switching to NIL in absence of optimal response (14). Ethics committees of all participating sites approved this study protocol. For the purposes of this *post-hoc* analysis, the cohort consisted of CML patients who had a completed HRQoL assessment at study entry (i.e., before study treatment start) and were randomly assigned to receive first-line treatment with NIL or IM between November 2016 and January 2021.Treatment responses were expressed on the International Scale (IS %) and were evaluated according to ELN2020 recommendations every 3 months (15). Early molecular response (EMR) was defined by BCR::ABL1 transcript levels ≤10% on the International Scale at 3 months of TKI treatment (5). HRQoL was assessed using the cancer-generic EORTC QLQ-C30 (16) and the disease specific EORTC QLQ-CML24 questionnaires (17). The QLQ-C30 consists of five functioning scales: physical, role, emotional, cognitive, and social; three symptom scales: fatigue, nausea/vomiting, and pain; six single-item scales: dyspnea, insomnia, appetite loss, constipation, diarrhea, and financial impact; and the global health status/QoL scale. The EORTC QLQ-CML24 consists of six scales: symptom burden, impact on worry/mood, impact on daily life, satisfaction with care and information, body image problems and satisfaction with social life). The main characteristics of newly diagnosed CML patients were summarized overall, using frequencies, proportions, means and standard deviations (SD), medians and interquartile ranges (IQR), depending on the type of variable. Given the exploratory nature of the study, all HRQoL scales were considered for their potential prognostic value for EMR. Univariate and multivariate logistic regression analyses were conducted to assess the prognostic effect of selected baseline socio-demographic and clinical characteristics along with HRQoL scales on EMR at 3 months. The starting univariate model included: sex, ECOG performance status, Sokal risk score, treatment arm, and all the HRQoL scales as continuous variables.

## Results

Overall, 448 newly diagnosed CML patients were enrolled and randomized to NIL and IM, 436 (97%) had a HRQoL assessment completed. Of them, 222 (51%) and 214 (49%) patients were randomly assigned to the NIL and IM arms, respectively. EMR was achieved in 190/202 (94%) and 151/194 (78%) patients, respectively (p<0.001). The median age of patients was 55 years (interquartile range, IQR 46-64) with a male predominance (57%). The Sokal score stratification identified 179 (41%), 185 (43%), and 70 (16%) as low, intermediate and high risk, respectively. According to the ELTS score, 274 (63%), 121 (28%), and 39 (9%), were classified as low, intermediate, and high risk, respectively. According to the EUTOS score, 375 (91%) were classified as low risk and 35 (9%) were classified as high risk. Details are reported in **Table 1**.

**Table 1:** Demographic and clinical characteristics of newly diagnosed CML patients at baseline.

Characteristic	Overall N = 436
Sex; n (%)	
Male	250 (57)
Female	186 (43)
Age; years	
Mean (SD)	54 (14)
Median (IQR)	55 (46 - 64)
Treatment arm; n (%)	
A - Imatinib	214 (49)
B - Nilotinib	222 (51)
ECOG performance status; n (%)	
0	375 (89)
≥1	46 (11)
Missing	15
Presence of any comorbidities; n (%)	
Yes	245 (57)
No	185 (43)
Missing	6
Sokal score; n (%)	
Low risk	179 (41)
Intermediate risk	185 (43)
High risk	70 (16)
Missing	2
ELTS score; n (%)	
Low risk	274 (63)
Intermediate risk	121 (28)
High risk	39 (9)
Missing	2
EUTOS score; n (%)	
Low risk	375 (91)
High risk	35 (9)
Missing	26

CML, Chronic Myeloid Leukemia; ECOG, Eastern Cooperative Oncology Group; ELTS, European Treatment and Outcome Study Long-term Survival; EUTOS, European Treatment and Outcome Study; SD, Standard Deviation; IQR, Interquartile Range.

In the univariate logistic regression analysis, EMR was significantly associated with Sokal risk stratification, treatment arm and the following patient-reported HRQoL: physical functioning, role functioning, social functioning scales of the EORTC-QLQ-C30 and body image problems of the EORTC-QLQ-CML24. In the multivariate analysis, the following variables retained a significance: the Sokal score stratification for the high category (OR 0.13, 95% CI 0.05-0.33, p<0.001) and intermediate risk (OR 0.43, 95% CI 0.18-0.96, p=0.046), the treatment arm with nilotinib (OR 4.61, 95% CI 2.31-9.88, p<0.001), and the role functioning scale of the EORTC-QLQ-C30 (OR 1.34, 95% CI 1.10-1.63, p<0.001). This latter indicates a 34% increase in the odds of achieving an EMR with every 10-point increase (i.e., improving) in the baseline role functioning scale. Details are reported in **Table 2**.

**Table 2:** Univariate and multivariate model on optimal vs non-optimal response achievement at 3 months from randomization.

Characteristic	Univariate			Multivariate		
	OR	95% CI	p-value	OR	95% CI	p-value
Sex						
Male	—	—				
Female	0.98	0.55, 1.76	>0.9	-	-	-
ECOG performance status; n (%)						
≥1	—	—		—	—	—
0	1.13	0.41, 2.66	0.8	—	—	—
Sokal score						
Low risk	—	—		—	—
High risk	0.12	0.05, 0.27	**<0.001**	0.13	0.05, 0.33	**<0.001**
Intermediate risk	0.35	0.15, 0.76	**0.011**	0.43	0.18, 0.96	**0.046**
Treatment Arm						
A-Imatinib	—	—		—	—	
B-Nilotinib	4.51	2.37, 9.22	**<0.001**	4.61	2.31, 9.88	**<0.001**
Presence of any comorbidities	1.26	0.71, 2.23	0.4	-	-	-
EORTC QLQ-C30 scales ^a^						
Global Health Status/QoL	1.10	0.90, 1.22	0.3	-	-	-
Physical Functioning	1.22	1.00, 1.48	**0.023**	-	-	-
Role Functioning	1.22	1.10, 1.34	**<0.001**	1.34	1.10, 1.63	**<0.001**
Emotional Functioning	1.00	0.90, 1.22	0.7	-	-	-
Cognitive Functioning	1.00	0.82, 1.22	0.8	-	-	-
Social Functioning	1.22	1.00, 1.34	**0.014**	-	-	-
Fatigue	0.90	0.82, 1.00	0.087	-	-	-
Nausea/Vomiting	1.00	0.74, 1.34	>0.9	1.00	0.74, 1.48	>0.99
Pain	1.00	0.82, 1.10	0.4	1.22	1.00, 1.48	0.070
Dyspnea	0.90	0.82, 1.00	0.053	-	-	-
Insomnia	1.00	0.82, 1.10	0.3	-	-	-
Appetite Loss	0.90	0.82, 1.10	0.4	-	-	-
Constipation	0.90	0.82, 1.00	0.056	-	-	-
Diarrhea	0.90	0.74, 1.10	0.2	-	-	-
Financial Difficulties	1.00	0.82, 1.22	0.8	-	-	-
EORTC QLQ-CML24 scales ^a^						
Symptom Burden Scale	0.82	0.66, 1.10	0.2	-	-	-
Impact On Worry/Mood Scale	0.82	0.74, 1.00	0.054	-	-	-
Impact On Daily Life Scale	0.90	0.74, 1.00	0.10	-	-	-
Satisfaction With Care And Information Scale	1.00	0.90, 1.10	0.7	-	-	-
Body Image Problems	0.90	0.74, 1.00	**0.016**	-	-	-
Satisfaction With Social Life	1.00	0.90, 1.10	0.6	-	-	-

OR, Odds Ratio; CI, Confidence interval; EORTC QLQ-C30, European Organisation for Research and Treatment of Cancer Quality of Life Questionnaire – Core 30; EORTC QLQ-CML24, European Organisation for Research and Treatment of Cancer Quality of Life Questionnaire for Chronic Myeloid Leukemia, 24 items. A stepwise regression has been used to select variable to include in the multivariate model. a. For questionnaires scales the OR is reported for every 10-point shift difference on the scale. Bold values indicate statistically significant p-values

The three CML prognostic scoring systems were able to disentangle the baseline HRQoL profiles of patients are reported in the (**Supplementary Materials S1**-**S3**).

Patients classified as high-risk by the Sokal risk score reported a significantly worse HRQoL profile than those classified as intermediate- and low-risk, as described by either worse functional impairments (i.e. lower global health status/QoL, physical and role functioning scale scores) or higher symptom severity (i.e. higher pain and dyspnea scale scores) and lower satisfaction with social life (disease-specific domains).

## Discussion

We found that the role functioning scale of the EORTC QLQ-C30, which broadly reflects the ability of patients to perform daily activities, provides independent prognostic information for the achievement of EMR. This finding supports the value of a more patient-centric approach at diagnostic workup of CML as it may offer unique information to better identify those patients who are more likely to achieve an important treatment milestone.

Similar to our result, a recent longitudinal HRQoL analysis, including pooled data from the BFORE trial, showed that clinical improvement was associated with variable effect on different dimensions of HRQoL, and patients achieving deep response (i.e. MR^5^) reported the greatest improvement in emotional well-being and leukemia specific domains (18).

A possible explanation of our findings could be the mediating role of adherence to therapy. That is, those patients who have higher ability to perform daily activities at diagnosis may be facilitated in fitting treatment schedule into their daily life, thereby adopting a more effective medication taking behavior and ultimately achieving an EMR at 3 months. However, we note that in our cohort, achievement of MMR at 6 months was not significantly associated with any baseline HRQoL domain (data not shown). In any case, given the exploratory nature of the analysis, our findings of the prognostic value of the role functioning scale of the EORTC QLQ-C30, should be further corroborated in future prospective studies.

Of note, none of the EORTC QLQ-CML24 domains emerged in the multivariate model, possibly suggesting that the EORTC QLQ-C30 alone, well captures key important aspects of patients with CML. As expected, treatment with nilotinib was significantly associated with a higher probability of achieving EMR, and this is in line with previous evidence on the higher efficacy of second-generation TKIs in inducing faster molecular responses compared to imatinib (19). Similarly, patients with lower Sokal risk scores had higher EMR rates, consistent with its prognostic role in this disease (20).

Our descriptive analysis investigating HRQoL profile according to the commonly used prognostic scoring systems, suggests that Sokal-risk score can help to disentangle some important HRQoL parameters of newly diagnosed patients.

In conclusion, our results suggest that assessment of HRQoL at diagnostic workup may offer independent prognostic information associated with a greater likelihood to achieve EMR.

## Author notes

Presented in part at the 29th European Hematology Association Congress (EHA 2024 Hybrid Congress) held in Madrid, Spain, June 13 to 16, 2024.

## Data Availability

The raw data supporting the conclusions of this article will be made available by the authors, without undue reservation.
