# Supplementary material for: The prognostic value of health-related quality of life for early molecular response in patients with chronic myeloid leukemia: analysis of the GIMEMA-SUSTRENIM trial
Source: Front Oncol. 2025 Aug 27;15:1645217. doi: 10.3389/fonc.2025.1645217 (PMC12420324; doi:10.3389/fonc.2025.1645217)
Supplement: Supplementary file 1 [file Table1.docx]

| **Supplementary table 1. HRQoL profile of newly diagnosed CML patient at baseline, by Sokal score** | | | | |  |
| --- | --- | --- | --- | --- | --- |
| **Characteristic** | **Low risk**,  N = 179 | **Intermediate risk**,  N = 185 | **High risk**,  N = 70 | **p-value** | |
| **EORTC QLQ-C30** |  |  |  |  | |
| **Functional Scale; Mean (SD)** |  |  |  |  | |
| Global Health Status / QoL | 73.9 (20.7) | 69.1 (22.1) | 66.2 (23.9) | **0.019** | |
| Physical Functioning | 92.1 (11.2) | 86.3 (16.3) | 85.0 (17.9) | **<0.001** | |
| Role Functioning | 89.7 (18.0) | 85.8 (22.4) | 78.6 (25.2) | **<0.001** | |
| Emotional Functioning | 77.6 (20.2) | 77.8 (20.6) | 77.8 (20.6) | >0.9 | |
| Cognitive Functioning | 91.7 (14.5) | 90.1 (16.0) | 91.0 (16.2) | 0.5 | |
| Social Functioning | 91.9 (15.4) | 90.5 (18.3) | 88.4 (17.5) | 0.2 | |
| **Symptom profile; Mean (SD)** |  |  |  |  | |
| Fatigue | 23.5 (23.3) | 24.7 (22.0) | 30.8 (25.2) | 0.080 | |
| Nausea / Vomiting | 4.3 (8.8) | 3.2 (9.1) | 4.3 (14.1) | 0.10 | |
| Pain | 11.2 (20.6) | 13.3 (20.2) | 17.9 (23.5) | **0.015** | |
| Dyspnea | 10.1 (18.0) | 12.9 (20.3) | 18.6 (22.4) | **0.010** | |
| Insomnia | 16.9 (25.6) | 22.5 (27.2) | 21.0 (27.3) | 0.076 | |
| Appetite Loss | 8.2 (19.3) | 7.6 (16.4) | 12.9 (22.2) | 0.11 | |
| Constipation | 8.0 (17.4) | 12.0 (21.2) | 13.8 (25.7) | 0.14 | |
| Diarrhea | 7.8 (15.8) | 6.9 (15.2) | 7.6 (15.2) | 0.8 | |
| Financial Difficulties | 5.8 (16.5) | 3.8 (13.6) | 6.3 (15.4) | 0.2 | |
|  |  |  |  |  | |
| **EORTC QLQ-CML24; Mean (SD)** |  |  |  |  | |
| Symptom Burden Scale | 12.9 (11.2) | 14.4 (11.4) | 15.0 (12.0) | 0.3 | |
| Impact On Worry/Mood Scale | 22.5 (17.7) | 22.7 (16.9) | 22.5 (18.1) | >0.9 | |
| Impact On Daily Life Scale | 15.0 (16.6) | 16.1 (20.3) | 15.2 (17.9) | >0.9 | |
| Satisfaction With Care And Information Scale | 78.1 (28.8) | 80.2 (27.8) | 74.6 (33.7) | 0.6 | |
| Body Image Problems | 10.3 (21.5) | 14.2 (22.8) | 11.8 (20.6) | 0.12 | |
| Satisfaction With Social Life | 72.6 (30.7) | 67.6 (30.8) | 61.3 (35.3) | **0.045** | |
| Abbreviations: HRQoL, Health-Related Quality of Life; CML, Chronic Myeloid Leukemia; EORTC QLQ-C30, European Organisation for Research and Treatment of Cancer Quality of Life Questionnaire – Core 30; EORTC QLQ-CML24, European Organisation for Research and Treatment of Cancer Quality of Life Questionnaire for Chronic Myeloid Leukemia, 24 items; SD, Standard deviations. | | | | |  |

| **Supplementary table 2. HRQoL profile of newly diagnosed CML patient at baseline, by ELTS score** | | | | |  |
| --- | --- | --- | --- | --- | --- |
| **Characteristic** | **Low risk**,  N = 274 | **Intermediate risk**,  N = 121 | **High risk**,  N = 39 | **p-value** | |
| **EORTC QLQ-C30** |  |  |  |  | |
| **Functional Scale; Mean (SD)** |  |  |  |  | |
| Global Health Status / QoL | 71.0 (21.7) | 69.9 (23.0) | 69.9 (21.3) | 0.8 | |
| Physical Functioning | 90.2 (13.3) | 85.0 (17.8) | 87.0 (15.1) | **0.013** | |
| Role Functioning | 88.4 (19.0) | 83.1 (25.0) | 80.3 (24.1) | **0.026** | |
| Emotional Functioning | 75.8 (21.1) | 81.5 (18.2) | 79.3 (20.5) | **0.031** | |
| Cognitive Functioning | 90.8 (15.2) | 90.9 (16.2) | 91.5 (15.2) | >0.9 | |
| Social Functioning | 91.3 (16.8) | 91.2 (17.0) | 85.0 (18.3) | **0.021** | |
| **Symptom profile; Mean (SD)** |  |  |  |  | |
| Fatigue | 25.1 (23.2) | 24.2 (23.1) | 29.3 (23.4) | 0.4 | |
| Nausea / Vomiting | 4.0 (9.5) | 3.2 (10.8) | 4.7 (10.1) | 0.5 | |
| Pain | 12.4 (21.1) | 14.6 (20.4) | 14.1 (22.8) | 0.2 | |
| Dyspnea | 12.2 (19.9) | 11.8 (19.2) | 17.9 (21.4) | 0.2 | |
| Insomnia | 19.6 (26.5) | 21.5 (27.2) | 17.9 (26.3) | 0.6 | |
| Appetite Loss | 9.0 (19.2) | 7.4 (16.9) | 10.3 (20.5) | 0.6 | |
| Constipation | 9.9 (19.9) | 11.7 (22.3) | 12.8 (21.1) | 0.5 | |
| Diarrhea | 8.3 (16.5) | 6.4 (13.9) | 4.3 (11.3) | 0.3 | |
| Financial Difficulties | 5.5 (16.9) | 4.1 (11.8) | 4.3 (11.3) | >0.9 | |
|  |  |  |  |  | |
| **EORTC QLQ-CML24; Mean (SD)** |  |  |  |  | |
| Symptom Burden Scale | 13.9 (11.5) | 13.8 (11.4) | 14.7 (11.0) | 0.8 | |
| Impact On Worry/Mood Scale | 24.0 (17.7) | 20.3 (16.9) | 19.9 (15.6) | 0.093 | |
| Impact On Daily Life Scale | 16.1 (17.8) | 15.1 (20.7) | 12.5 (15.5) | 0.2 | |
| Satisfaction With Care And Information Scale | 79.3 (28.2) | 76.8 (31.4) | 77.5 (30.0) | 0.9 | |
| Body Image Problems | 12.3 (22.2) | 12.6 (23.1) | 10.5 (15.7) | >0.9 | |
| Satisfaction With Social Life | 70.3 (30.0) | 66.7 (33.5) | 62.2 (37.0) | 0.5 | |
| Abbreviations: HRQoL, Health-Related Quality of Life; CML, Chronic Myeloid Leukemia; EORTC QLQ-C30, European Organisation for Research and Treatment of Cancer Quality of Life Questionnaire – Core 30; EORTC QLQ-CML24, European Organisation for Research and Treatment of Cancer Quality of Life Questionnaire for Chronic Myeloid Leukemia, 24 items; ELTS, European Treatment and Outcome Study Long-term Survival; SD, Standard deviations. | | | | |  |

| **Supplementary table 3. HRQoL profile of newly diagnosed CML patient at baseline, by EUTOS score** | | | |  |
| --- | --- | --- | --- | --- |
| **Characteristic** | **Low risk**,  N = 375 | **High risk**,  N = 35 | **p-value** | |
| **EORTC QLQ-C30** |  |  |  | |
| **Functional Scale; Mean (SD)** |  |  |  | |
| Global Health Status / QoL | 70.3 (22.0) | 74.5 (20.1) | 0.3 | |
| Physical Functioning | 88.1 (15.5) | 89.9 (10.0) | 0.8 | |
| Role Functioning | 86.4 (21.7) | 83.3 (16.7) | 0.065 | |
| Emotional Functioning | 77.6 (20.5) | 78.2 (18.7) | >0.9 | |
| Cognitive Functioning | 91.0 (15.5) | 92.9 (10.9) | 0.9 | |
| Social Functioning | 91.0 (17.0) | 87.1 (15.7) | **0.028** | |
| **Symptom profile; Mean (SD)** |  |  |  | |
| Fatigue | 25.1 (23.6) | 26.7 (20.4) | 0.4 | |
| Nausea / Vomiting | 4.0 (10.1) | 4.3 (11.0) | 0.7 | |
| Pain | 13.3 (21.1) | 12.9 (19.4) | 0.8 | |
| Dyspnea | 12.7 (20.0) | 14.3 (20.3) | 0.6 | |
| Insomnia | 20.6 (27.2) | 11.4 (18.0) | 0.077 | |
| Appetite Loss | 8.3 (18.3) | 9.5 (17.3) | 0.5 | |
| Constipation | 10.3 (20.6) | 13.3 (23.2) | 0.4 | |
| Diarrhea | 7.6 (15.8) | 7.6 (14.2) | 0.8 | |
| Financial Difficulties | 5.1 (15.5) | 4.8 (14.3) | >0.9 | |
|  |  |  |  | |
| **EORTC QLQ-CML24; Mean (SD)** |  |  |  | |
| Symptom Burden Scale | 13.9 (11.6) | 14.3 (9.5) | 0.5 | |
| Impact On Worry/Mood Scale | 22.9 (17.6) | 20.7 (16.2) | 0.5 | |
| Impact On Daily Life Scale | 16.0 (19.0) | 13.9 (15.0) | 0.8 | |
| Satisfaction With Care And Information Scale | 78.8 (28.7) | 88.2 (17.2) | 0.11 | |
| Body Image Problems | 13.1 (22.9) | 7.8 (14.4) | 0.3 | |
| Satisfaction With Social Life | 68.5 (31.4) | 72.7 (26.9) | 0.6 | |
| Abbreviations: HRQoL, Health-Related Quality of Life; CML, Chronic Myeloid Leukemia; EORTC QLQ-C30, European Organisation for Research and Treatment of Cancer Quality of Life Questionnaire – Core 30; EORTC QLQ-CML24, European Organisation for Research and Treatment of Cancer Quality of Life Questionnaire for Chronic Myeloid Leukemia, 24 items; EUTOS, European Treatment and Outcome Study; SD, Standard deviations. | | | |  |
